# Real-World Impact of Immune Checkpoint Inhibitors in Metastatic Uveal Melanoma

**DOI:** 10.3390/cancers11101489

**Published:** 2019-10-03

**Authors:** Kalijn Fredrike Bol, Eva Ellebaek, Lise Hoejberg, Mette Marie Bagger, Mathilde Skaarup Larsen, Tobias Wirenfeldt Klausen, Ulrich Heide Køhler, Henrik Schmidt, Lars Bastholt, Jens Folke Kiilgaard, Marco Donia, Inge Marie Svane

**Affiliations:** 1 Department of Oncology, National Center for Cancer Immune Therapy, Herlev Hospital, Copenhagen University Hospital, 2730 Herlev, Denmark; Kalijn.Fredrike.Bol@regionh.dk (K.F.B.); Eva.Ellebaek.Steensgaard@regionh.dk (E.E.); Tobias.Wirenfeldt.Klausen@regionh.dk (T.W.K.); Marco.Donia@regionh.dk (M.D.); 2 Department of Oncology, Odense University Hospital, 5000 Odense, Denmark; Lise.Hoejberg@rsyd.dk (L.H.); uhk@braintrust-consult.dk (U.H.K.); Lars.Bastholt@rsyd.dk (L.B.); 3 Department of Ophthalmology, Rigshospitalet, Copenhagen University Hospital, 2100 Copenhagen, Denmark; Mette.Marie.Bagger.01@regionh.dk (M.M.B.); Jens.Folke.Kiilgaard@regionh.dk (J.F.K.); 4 Department of Pathology, Herlev Hospital, Copenhagen University Hospital, 2730 Herlev, Denmark; Mathilde.Skaarup.Larsen@regionh.dk; 5 Department of Oncology, Aarhus University Hospital, 8200 Aarhus, Denmark; henrschm@rm.dk

**Keywords:** uveal melanoma, immunotherapy, immune checkpoint inhibitors, real-world data

## Abstract

Uveal melanoma (UM) is the most common intraocular malignancy in adults and shows a high rate of metastatic spread. As randomized clinical trials with immune checkpoint inhibitors (ICI) have not been performed in patients with metastatic UM, we analyzed the real-world outcomes in a nationwide population-based study. Clinical data of patients with UM were extracted from the Danish Metastatic Melanoma database, a nationwide database containing unselected records of patients diagnosed with metastatic melanoma in Denmark. Survival before (pre-ICI, *n* = 32) and after (post-ICI, *n* = 94) the approval of first-line treatment with ICI was analyzed. A partial response to first-line treatment was observed in 7% of patients treated with anti-programmed cell death protein (PD)-1 monotherapy and in 21% with combined anti-cytotoxic T lymphocyte antigen (CTLA)-4 plus anti-PD-1 therapy. Median progression-free survival was 2.5 months for patients treated in the pre-ICI era compared to 3.5 months in the post-ICI era (hazard ratio (HR) 0.43; 95% confidence interval (CI) 0.28–0.67; *p* < 0.001). The estimated one-year overall survival rate increased from 25.0% to 41.9% and the median overall survival improved from 7.8 months to 10.0 months, respectively (HR 0.52; 95% CI 0.34–0.79; *p* = 0.003). Thus, the introduction of ICI as first-line treatment appears to have significantly improved the real-world survival of patients with metastatic UM, despite relatively low response rates compared to cutaneous melanoma. With the lack of therapies proven effective in randomized trials, these data support the current treatment with ICI in patients with metastatic UM.

## 1. Introduction

Uveal melanoma (UM) is the most common intraocular malignancy in adults and it arises in the melanocytes in the uveal tract of the eye, mainly in the choroid. With an incidence of 2–8 per million persons per year, it remains a rare disease and only constitutes ~3% of patients with metastatic melanoma [1,2]. UM is clinically and genetically distinct from cutaneous melanoma (CM), e.g., different genes are mutated and where in CM lymphogenic spread is common, it does not occur in UM. Despite adequate local treatment, approximately half of the patients with a primary UM will develop metastases, with a strong predilection for metastatic spread to the liver [3,4].

At present, no systemic treatment has proven to result in survival benefit in patients with metastatic UM [5]. With the lack of effective standard-of-care treatment for metastatic UM, most guidelines recommend participation in clinical trials [6,7]. However, patients with metastatic UM are often excluded from melanoma trials and access to UM-specific clinical trials is limited. As a result, most patients are treated with the drugs approved for metastatic CM, despite lack of evidence for efficacy in metastatic UM. Thus, following drug approvals in CM over the past decade, the first-line treatment shifted from chemotherapy (temozolomide or dacarbazine) to immunotherapy, with the immune checkpoint inhibitors (ICI) such as anti-cytotoxic T lymphocyte antigen (CTLA)-4 (ipilimumab) or anti-programmed cell death protein (PD)-1 antibodies (pembrolizumab or nivolumab) in multiple countries. In contrast to CM, BRAF inhibitors are not part of the treatment arsenal of patients with metastatic UM as BRAF mutations do not occur in UM [8,9]. Targeted therapy with MEK inhibitors is one of the few systemic treatments tested in a phase III clinical trial in UM but failed to show clinical benefit in combination with chemotherapy in the randomized setting [10].

In the last decade, the success of ICI has revolutionized the treatment of patients with metastatic melanoma and numerous other tumor types. In 2011, ipilimumab was the first approved ICI for melanoma, showing an objective response rate (ORR) of 11–15% in patients with metastatic CM [11,12]. However, in UM, three single-arm phase II clinical trials, multiple expanded access programs and retrospective analyses of clinical databases consistently showed ORR of 0–8% [13,14,15,16,17,18,19,20]. On a similar note, while monotherapy with anti-PD-1 antibodies displayed an ORR of 33–40% in CM [21,22], ORR in retrospective analyses of patient cohorts with UM did not exceed 8% [23,24,25,26,27,28,29,30]. The same trend is seen with combined of ipilimumab/nivolumab, reaching an ORR of 58% in CM [31] and 0-17% in two small retrospective cohorts and a single-arm phase II clinical trial in patient with metastatic UM [29,32,33].

Overall, substantially lower response rates on ICI are reported in metastatic UM compared to CM. Still, data is limited, and randomized clinical trials have not been performed. Most data originate from small, retrospective cohorts and largely consist of highly selected patients with no comparison to other treatments. Therefore, we analyzed the real-world outcomes of treatment with ICI and chemotherapy in unselected patients with metastatic UM in a nationwide, population-based study.

## 2. Materials and Methods

### 2.1. Patient Population and Databases

We performed a nationwide, retrospective population-based study of all registered patients with metastatic UM in Denmark. All patients with metastatic melanoma that are not amenable for local treatment or progressed after local treatment of metastases are referred to the department of clinical oncology in one of the three reference centers (Aarhus, Odense and Herlev University Hospitals). Patients with an initial oncological evaluation between 1 January 2011 and 31 December 2018 were included. All records were retrieved from the Danish Metastatic Melanoma database (DAMMED), which has an estimated coverage of >95% of metastatic melanoma patients in Denmark. The database was locked on May 6, 2019. As the records were categorized as ocular melanoma in the Danish Metastatic Melanoma database, diagnosis of primary UM was confirmed via the Copenhagen Epidemiological Uveal Melanoma Study database (COEUS) [34]. This database contains all patients with a primary UM in Denmark from 1943 onward (>3900 patients), independent of whether pathology of the primary is available. Patients with metastasis from a primary conjunctival melanoma (*n* = 2) or orbital melanoma (*n* = 1) were excluded from the analysis. The Danish Metastatic Melanoma Database (2011-41-6802) and the Copenhagen Epidemiological Uveal Melanoma Study database (2016-41-4897) were approved by the Danish Data Protection Agency in 2011 and 2016, respectively.

### 2.2. Treatment and Response

Patients were treated according to best practice which consisted of chemotherapy (temozolomide), immunotherapy (ipilimumab, pembrolizumab or combined ipilimumab/nivolumab) or best supportive care. Few patients were included in clinical trials. Patients were analyzed according to actual received treatment or in time periods depending on date of drug approval for first-line treatment in melanoma in Denmark: pre-ICI era (2011–2013) versus post-ICI era (2014–2018).

Tumor response was assessed according to the Response Evaluation Criteria in Solid Tumor (RECIST) guidelines [35]. Durable stable disease (SD) was defined as stable disease for at least 24 weeks. The ORR was defined as the proportion of patients who achieved a complete response (CR) or partial response (PR). The disease control rate was defined as the proportion of patients who achieved a CR, PR or SD.

Progression-free survival (PFS) was defined as the time from initiation of systemic treatment to the date of documented disease progression or last follow-up. Overall survival (OS) was defined as the time from initiation of first-line systemic treatment to death or last follow-up. In patients who did not receive any systemic treatment, OS was calculated from the date of initial oncological evaluation.

### 2.3. Statistical Analysis

Statistical significance of baseline characteristics was evaluated using chi-square tests. Survival was estimated using the Kaplan-Meier method and compared using the log-rank test. Follow-up duration was estimated using the Kaplan-Meier method with the time from initiation of first-line treatment, or date of initial oncological evaluation in patients who did not receive systemic treatment, to date of last follow-up and censored for death. Hazard ratios (HR) and corresponding 95% confidence intervals (CI) were calculated with the Cox proportional hazards model. All *p*-values were two-sided and *p*-values < 0.05 were considered statistically significant. SPSS Statistics version 25.0 software (IBM Corporation, Armonk, NY, USA) was used for statistical analysis.

## 3. Results

### 3.1. Patient Population

A total of 126 patients with metastatic UM, referred for systemic treatment of distant metastases between 2011 and 2018, were retrieved from the Danish Metastatic Melanoma database. At the time of data analysis, median follow-up was 23 months and 19% of patients were still alive with a minimum follow-up time of 5 months. Thirty-two patients (25%) were treated before approval of the first ICI as first-line treatment, pre-ICI era (2011–2013), and 94 patients (75%) were treated after first-line treatment with ICI became available, post-ICI era (2014–2018). The baseline characteristics of all metastatic UM patients are summarized in Table 1, including the known prognostic factors age, lactate dehydrogenase (LDH), Eastern Cooperative Oncology Group (ECOG) performance status and time to metastatic disease. No statistically significant differences were present between the two groups. In seven patients a liver resection was recorded before start of systemic treatment; four patients showed recurrent disease within six months, one patient after one year and two patients after a bit more than two years.

### 3.2. First-Line Treatment in the pre-ICI and post-ICI Era

**Figure 1:**
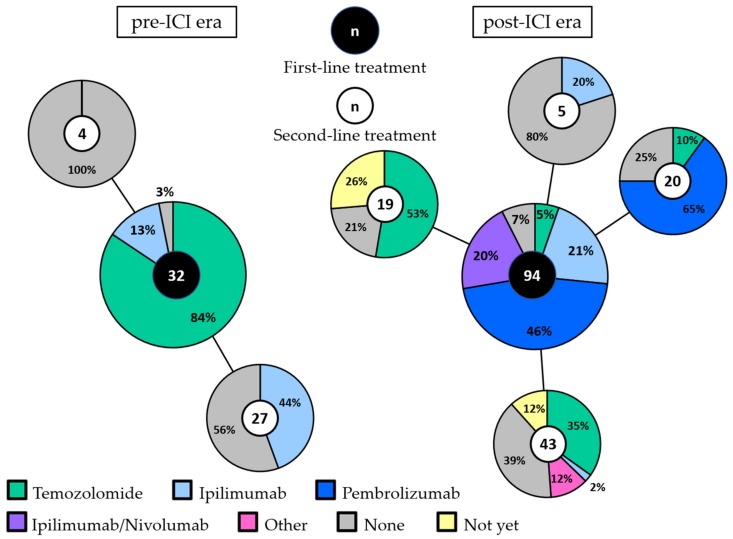
First-line and second-line treatment in the pre-ICI and post-ICI era. The large pie charts represent the first-line treatment in the pre-ICI era (left) and the post-ICI era (right). Second-line treatment is shown per first-line treatment in the connected smaller pie charts. The total number of patients in each pie chart is depicted in the middle. Patients that are still alive, either with ongoing partial response or stable disease to first-line treatment or with progression but did not receive second-line treatment yet, are depicted in yellow (“not yet”). Abbreviations: ICI, immune checkpoint inhibitor.

In the pre-ICI era, first-line treatment consisted of chemotherapy (temozolomide) in the majority of patients (84%: Figure 1). Four patients received ipilimumab within a clinical trial as first-line treatment. Twelve patients treated with first-line chemotherapy received ipilimumab as second-line treatment. None of the patients received anti-PD-1 antibodies.

In the post-ICI era, only five patients (5%) received chemotherapy as first-line treatment. Ipilimumab, pembrolizumab and combined ipilimumab/nivolumab were given as first-line treatment in 21%, 46% and 20% of patients, respectively. No systemic treatment was administered to one patient in the pre-ICI era (3%) and to seven patients in the post-ICI era (7%). All but one of these patients had an elevated LDH and/or ECOG performance status of 2 or 3.

### 3.3. Survival Per Treatment Era

**Figure 2:**
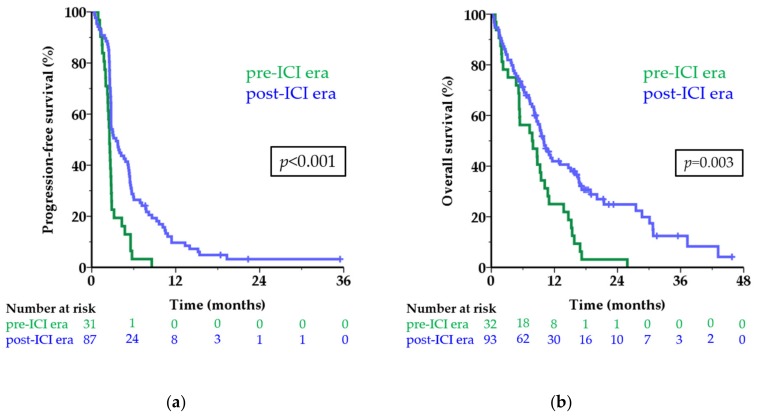
Survival before and after approval of first-line treatment with ICI. Kaplan-Meier curves showing progression-free survival (**a**) and overall survival (**b**) in the pre-ICI era (green line) and post-ICI era (blue line) in months for patients with metastatic uveal melanoma. The numbers below the figures denote the number of patients ‘at risk’ in each group. Abbreviations: ICI, immune checkpoint inhibitor.

The median PFS of first-line treatment in the pre-ICI era was 2.5 months versus 3.5 months in the post-ICI era (HR 0.43; 95 CI 0.28–0.67; *p* < 0.001; Figure 2a). The six-month PFS rate was 3.2% and 27.6%, respectively. Among patients in the post-ICI era, 9.7% were still free of progression one year after start of treatment versus none in the pre-ICI era.

The median OS was 7.8 months in the pre-ICI era versus 10.0 months in the post-ICI era (HR 0.52; 95% CI 0.34–0.79; *p* = 0.003; Figure 2b). The one-year OS rate increased from 25.0% to 41.9% after the introduction of first-line treatment with ICI. When excluding patients who did not receive any systemic treatment the difference in survival remained similar (median OS 7.9 versus 10.1 months; one-year OS rate 25.8% versus 42.9%; *p* = 0.002).

### 3.4. Objective Response Rates and Survival per First-Line Treatment

Independent of treatment eras, first-line treatment consisted of temozolomide in 32, ipilimumab in 24, pembrolizumab in 43 and a combination ipilimumab/nivolumab in 19 patients (Table 2). A PR was observed in three patients (7.0%) treated with pembrolizumab and in four patients (21.1%) treated with combined ipilimumab/nivolumab. At time of data analysis, four patients had an ongoing PR, with follow-up ranging from 8 to 35 months. Three patients showed progressive disease after an initial PR, after 11, 14 (combined ipilimumab/nivolumab) and 15 months (pembrolizumab), respectively. No objective responses were observed in patients treated with temozolomide or ipilimumab and no CR were achieved in the entire cohort. The disease control rate, including stable disease with at least 24 weeks duration, was 6.3% with temozolomide, 25.0% with ipilimumab, 34.9% with pembrolizumab and 31.6% with combined ipilimumab/nivolumab.

Eight patients did not receive any systemic treatment; they had a very short median OS of only 1.5 months, with two patients surviving longer than one year. Patients treated with any first-line ICI showed significantly longer survival, both PFS and OS, compared to chemotherapy (Table 2; Figure S1). As seen in CM, the benefit of treatment with ICI in UM is hardly displayed by comparing the median survival times, but the subset of patients achieving long-term benefit is appreciated in the tail of the curve and shown by the improvement in survival rates. Interestingly, all patients in the tails of the curves in Figure 2 (defined as a PFS of 12 months or longer and an OS of 24 months or longer) received ICI as first-line treatment.

### 3.5. Retreatment and Second-Line Treatment

Three patients received retreatment after initial stable disease. Renewed disease control was only obtained in one patient who received pembrolizumab for progressive disease which occurred three months after stopping combined ipilimumab/nivolumab because of toxicity. The two other patients, one retreated with ipilimumab, the other treated with nivolumab after progression on pembrolizumab, showed PD within 12 weeks of retreatment.

In total, 27 patients received temozolomide as second-line treatment, 14 received ipilimumab and 13 received pembrolizumab. As with first-line treatment, no objective responses were observed in patients treated with temozolomide or ipilimumab in second line. Two patients obtained a PR on pembrolizumab as second-line treatment (15.4%), of which one is ongoing for more than 3.5 years.

## 4. Discussion

Randomized clinical trials with ICI have not been performed in metastatic UM patients. Still, numerous metastatic UM patients are currently treated with ICI outside of clinical trials. In this nationwide population-based study, we showed that objective responses were observed in 7% and 21% of patients treated with first-line pembrolizumab or combined ipilimumab/nivolumab, respectively. Furthermore, with the introduction of ICI as first-line treatment the median OS increased from 7.8 to 10.0 months and a rise in one-year OS rate from 25.0% to 41.9% was observed. Compared to clinical trials, real-world data generally show lower response rates and poorer survival as the unselected patient population usually has inferior baseline characteristics than trial participants [36]. In our nationwide population-based study, 12% of the patients had an ECOG performance score of ≥2 and 64% of the patients had an increased LDH, while only 6% of patients did not receive any systemic treatment. As expected, the baseline characteristics at start of systemic treatment of our real-world population were slightly to significantly worse compared to previous reports on ICI in metastatic UM, i.e., the poor prognostic factors (higher ECOG performance score and higher LDH) were present more frequently in our cohort. Surprisingly, the response rates to pembrolizumab and combined ipilimumab/nivolumab in our population-based study are among the highest described in literature, as are the median PFS of 3.5 months in the post-ICI era and the median PFS per treatment of 4.8 months for pembrolizumab and 3.7 months for combined ipilimumab/nivolumab. A possible explanation for the higher response rate of pembrolizumab found in our real-world population compared to literature might be chance or the difference in treatment line; in our study, we analyzed response to first-line treatment with ICI only, while in other reports, the majority of patients did receive ICI after previous treatment. An exception is the largest published cohort (*n* = 86) of which 67% of patients were treated with anti-PD-1 antibodies in first line, showing an ORR of 4.7% and a median PFS between 2.8 and 3.1 months [29]. Although responses to first-line treatment are likely to be substantially higher than to later lines of treatment, we observed a PR in 2 out of 13 patients (15.4%) treated with pembrolizumab in second line. However, the ORR might be overestimated due to the small number of patients receiving pembrolizumab in second line. Nonetheless, our ORR to combined ipilimumab/nivolumab is also higher than in a single-arm phase 2 clinical trial with the combination as first-line treatment, showing an ORR of 12% in 50 evaluable metastatic UM patients with more favorable baseline characteristics [33]. However, the group of patients treated with combined ipilimumab/nivolumab in our cohort consists of only 19 patients; thus, the response rate of 12% lies within the confidence interval.

No clinical trials have been performed to compare ICI with chemotherapy in metastatic UM. Mignard et al. retrospectively analyzed patients with metastatic UM treated with chemotherapy or ICI. No objective responses were observed with ICI and survival did not significantly differ between ICI and chemotherapy (median OS 13.4 versus 11.0 months; HR 0.88; *p* = 0.48) [37]. The lack of benefit of ICI in this cohort, in contrast to our data, might be explained by fewer treatment-naïve patients, less patients receiving anti-PD-1 antibodies and exclusion of patients treated with combined ipilimumab/nivolumab. Another retrospective meta-analysis showed a median OS of 10.9 months (0.91 years) for patients treated with chemotherapy and a significantly worse median OS of 7.1 months (0.59 years) for patients treated with ICI [5]. However, approximately 50% of patients received chemotherapy as first-line and <10% of patients were treated with ICI as first line, which has likely biased the data. In addition, most patients in the ICI group received ipilimumab while only 31% received the more effective ICI targeting PD-1 (versus 66% in our cohort).

Our study has some limitations inherent to its retrospective design and rarity of the disease. For example, ECOG performance score and LDH were not documented in about half of the patients in the pre-ICI era. Although, known baseline characteristics are well balanced between the pre-ICI and post-ICI era, the missing data might conceal some differences. One disbalance between the groups is present as the liver resections were all performed on patients in the post-ICI era. However, only three patients obtained clinical benefit from the surgery and are unlikely to have biased the results as two showed progressive disease on first-line treatment and only one patient showed stable disease for approximately 10 months on pembrolizumab. Furthermore, the treatments in the post-ICI era are diverse and subgroups per treatment remain too small to draw firm conclusions on response rates, despite representing one of the biggest cohorts of metastatic UM patients treated with ICI.

Nevertheless, the analysis of our unselected patient population is likely to underestimate the benefit of ICI treatment in metastatic UM. Survival differences between the treatments might be undervalued as 50% of patients in the pre-ICI era received ipilimumab either as first-line treatment within a clinical trial or as second-line treatment. If only patients treated with temozolomide in the pre-ICI era and patients treated with ICI in the post-ICI era are analyzed, the difference in OS increases (median OS 5.3 versus 10.6 months; HR 0.34; 95% CI 0.19–0.62; *p* < 0.001). In addition, after physicians became familiar with pembrolizumab, with its favorable toxicity profile, a higher proportion of patients were treated; in the last two years, all patients with a poor ECOG performance score received systemic treatment. Still, comparing the pre-ICI and post-ICI era seems the fairest comparison possible, compared to per treatment, as choice of treatment might be dependent on patient characteristics, e.g., less fit patients might not get treated with combined ipilimumab/nivolumab but with pembrolizumab monotherapy to reduce the risk of toxicity. As no major changes were made in the treatment of primary UM in the last decade and no effective adjuvant treatment is available, the bias in time (2011–2013 versus 2014–2018) is considered to be absent or minimal. This is substantiated by literature as no association between survival and time was present over the past few decades [5]. Furthermore, the durable benefit of ICI treatment in metastatic UM is substantiated by the observation that all patients in the tails of the survival curves were treated with first-line ICI.

The awaited results of a phase II clinical trial with the combination as any line of treatment (NCT01585194) and the full results of combination as first-line treatment (NCT02626962) might give more insight in the response rate to combined ipilimumab/nivolumab. However, no randomization takes place in these trials so comparison with other treatments will be difficult and it is unlikely that a randomized clinical trial with ICI will be performed in metastatic UM. Besides clinical trials, further investigation to determine predictive biomarkers for response to ICI is warranted to select patients for ICI treatment, e.g., specific germline mutations [38], tumor mutational burden and the immune infiltrate in the tumor microenvironment. Overall, available data show lower response rates with ICI compared to metastatic CM; however, a significant proportion of metastatic UM patients seem to clinically benefit from ICI treatment, especially from combined ipilimumab/nivolumab.

## 5. Conclusions

The introduction of ICI as first-line treatment appears to have significantly improved the real-world survival of patients with metastatic UM. Despite relatively low response rates, the response rate in patients treated with combined ipilimumab/nivolumab is still promising. Thus, with the lack of therapies proven effective in randomized trials, these data support the current treatment with combination ICI in patients with metastatic UM if participation in a clinical trial is not possible.

## Figures and Tables

**Table 1:** Baseline characteristics.

Characteristic		Pre-ICI era *n* = 32 (%)	Post-ICI era *n* = 94 (%)
Age	median (range)	62 (34–89)	65 (22–87)
Sex	male female	14 (43.8%) 18 (56.3%)	47 (50.0%) 47 (50.0%)
AJCC stage at diagnosis	stage I stage IIA stage IIB stage IIIA stage IIIB stage IIIC stage IV unknown	1 (3.1%) 11 (34.4%) 11 (34.4%) 6 (18.8%) 2 (6.3%) 0 (0%) 1 (3.1%) 0 (0%)	7 (7.4%) 25 (26.6%) 24 (25.5%) 20 (21.3%) 10 (10.6%) 3 (3.2%) 4 (4.3%) 1 (1.1%)
ECOG performance status	0 1 2 3 unknown	11 (57.9%) 5 (26.3%) 2 (10.5%) 1 (5.3%) 13	53 (60.2%) 25 (28.4%) 9 (10.2%) 1 (1.1%) 6
Metastatic sites	liver only extrahepatic only liver + extrahepatic	15 (46.9%) 2 (6.3%) 15 (46.9%)	39 (41.5%) 8 (8.5%) 47 (50.0%)
LDH	LDH ≤ ULN LDH 1-2× ULN LDH > 2× ULN unknown	4 (25.0%) 6 (37.5%) 6 (37.5%) 16	34 (38.2%) 33 (37.1%) 22 (24.7%) 5
Time from primary diagnosis to metastatic disease	<1 year 1–3 years >3 years	3 (9.4%) 10 (31.3%) 19 (59.4%)	22 (23.4%) 27 (28.7%) 45 (47.9%)
Time to start systemic treatment *	<6 months 6–12 months >12 months	30 (96.7%) 1 (3.2%) 0 (0%)	75 (86.2%) 7 (8.0%) 5 (5.7%)

* Calculated from the diagnosis of metastatic disease, excluding patients who did not receive systemic treatment. AJCC, American Joint Committee on Cancer; ECOG performance status, Eastern cooperative oncology group performance status; ICI, immune checkpoint inhibitor: LDH, lactate dehydrogenase; ULN, upper limit of normal.

**Table 2:** Response per first-line treatment.

	Temozolomide *n* = 32 (%)	Ipilimumab *n* = 24 (%)	Pembrolizumab *n* = 43 (%)	Ipilimumab/Nivolumab *n* = 19 (%)
CR	0 (0%)	0 (0%)	0 (0%)	0 (0%)
PR	0 (0%)	0 (0%)	3 (7.0%)	4 (21.1%)
SD ≥ 24 weeks	2 (6.3%)	6 (25.0%)	12 (27.9%)	2 (10.5%)
PD	30 (93.4%)	18 (75.0%)	28 (65.1%)	13 (68.4%)
PFS, median	2.5	3.0	4.8	3.7
6-month PFS rate	3.1%	16.7%	32.6%	31.6%
OS, median	5.7	9.9	10.3	18.9
1-year OS rate	18.8%	50.0%	38.7%	57.6%

CR, complete response; OS, overall survival (in months); PD, progressive disease; PFS, progression-free survival (in months); PR, partial response; SD, stable disease.

## Supplementary Materials

The following is available online at https://www.mdpi.com/2072-6694/11/10/1489/s1, Figure S1: Survival per first-line treatment.

## Abbreviations

The following abbreviations are used in this manuscript:

AJCC:	American Joint Committee on Cancer
CI:	confidence intervals
CM:	cutaneous melanoma
CR:	complete response
ECOG:	Eastern cooperative oncology group
HR:	hazard ratio
ICI:	immune checkpoint inhibitors
LDH:	lactate dehydrogenase
OS:	overall survival
ORR:	objective response rate
PFS:	progression-free survival
PR:	partial response
SD:	stable disease
ULN:	upper limit of normal
UM:	uveal melanoma
