# Supplementary material for: Real-World Impact of Immune Checkpoint Inhibitors in Metastatic Uveal Melanoma
Source: Cancers (Basel). 2019 Oct 3;11(10):1489. doi: 10.3390/cancers11101489 (PMC6826482; doi:10.3390/cancers11101489)
Supplement: Supplementary file 1 [file cancers-11-01489-s001.pdf]

Supplementary Materials

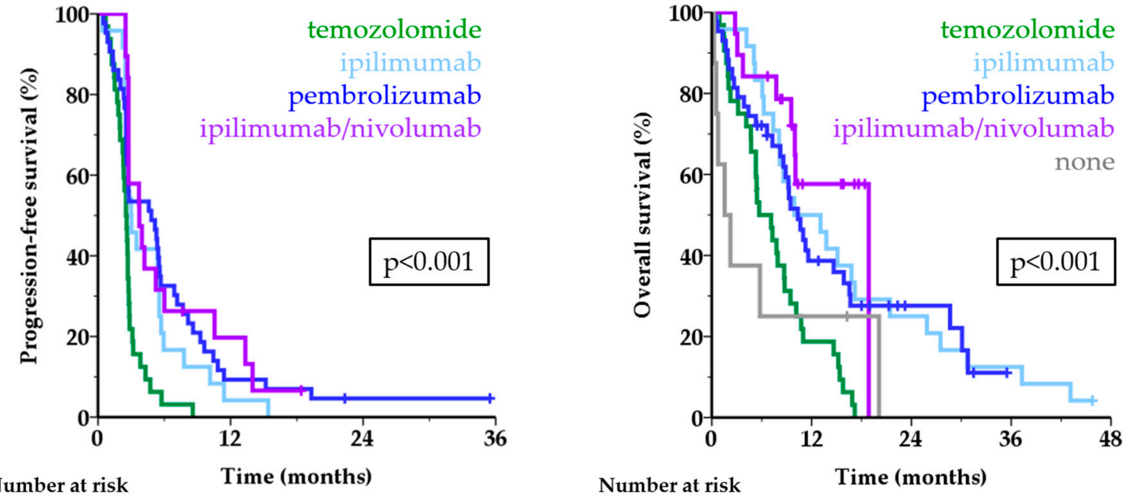

Number at risk							
temo	32	1	0	0	0	0	0
ipi	24	4	1	0	0	0	0
pembro	43	14	4	3	1	1	0
ipi/nivo	19	6	3	1	0	0	0

(**a**)

Number at risk									
temo	32	16	6	0	0	0	0	0	0
ipi	24	20	12	7	6	4	3	2	0
pembro	42	29	15	8	5	3	0	0	0
ipi/nivo	19	13	4	2	0	0	0	0	0
none	8	2	2	1	0	0	0	0	0

(**b**)

Figure S1. Survival per first-line treatment**.** Kaplan-Meier curves showing progression-free survival (**a**) and overall survival (**b**) per first-line treatment (temozolomide in green, ipilimumab in light blue, pembrolizumab in dark blue, combined ipilimumab/nivolumab in purple, no systemic treatment in grey) in months for metastatic uveal melanoma patients. The numbers below the figures denote the number of patients ‘at risk’ in each group.
